# Development of a Cumulative Exposure Index (CEI) for Manganese and Comparison with Bone Manganese and Other Biomarkers of Manganese Exposure

**DOI:** 10.3390/ijerph15071341

**Published:** 2018-06-26

**Authors:** Danelle Rolle-McFarland, Yingzi Liu, Jieqiong Zhou, Farshad Mostafaei, Yuanzhong Zhou, Yan Li, Quiyan Fan, Wei Zheng, Linda H. Nie, Ellen M. Wells

**Affiliations:** 1 School of Health Sciences, Purdue University, West Lafayette, IN 47907, USA; Danelle.Rolle-Mcfarland@moffitt.org (D.R.-M.); yliu@wkhs.com (Y.L.); jieqiong.zhou@yale.edu (J.Z.); fmostafaei@mcw.edu (F.M.); wzheng@purdue.edu (W.Z.); hnie@purdue.edu (L.H.N.); 2 Department of Cancer Epidemiology, H. Lee Moffitt Cancer Center and Research Institute, Tampa, FL 33612, USA; 3 Willis-Knighton Cancer Center, Shreveport, LA 71103, USA; 4 Department of Radiation Oncology, Medical College of Wisconsin, Milwaukee, WI 53226, USA; 5 School of Public Health, Zunyi Medical University, Zunyi 563003, Guizhou, China; zhouyuanzhong@163.com (Y.Z.); liyan067321@sina.com (Y.L.); 6 Zunyi Medical and Pharmaceutical College, Zunyi 563003, Guizhou, China; fanqy@zunyiyizhuan.cn

**Keywords:** manganese, cumulative exposure index, biomarkers, blood manganese, fingernail manganese, bone manganese, in vivo neutron activation analysis

## Abstract

Manganese (Mn) exposure can result in parkinsonism. However, understanding of manganese neurotoxicity has been limited by the lack of a cumulative Mn biomarker. Therefore, the current goal was to develop Mn cumulative exposure indices (MnCEI), an established method to estimate cumulative exposure, and determine associations of MnCEI with blood Mn (BMn), fingernail Mn (FMn), and bone Mn (BnMn). We completed a cross-sectional study of 60 male Chinese workers. Self-reported occupational history was used to create two MnCEIs reflecting the previous 16 years (MnCEI_16_) and total work history (MnCEI_TOT_). An in vivo neutron activation analysis system was used to quantify BnMn. BMn and FMn were measured using ICP-MS. Mean (standard deviation) MnCEI_TOT _and MnCEI_16 _were 37.5 (22.0) and 25.0 (11.3), respectively. Median (interquartile range) BMn, FMn, and BnMn were 14.1 (4.0) μg/L, 13.5 (58.5) μg/g, and 2.6 (7.2) μg/g dry bone, respectively. MnCEI_16 _was significantly correlated with FMn (Spearman’s *ρ *= 0.44; *p *= 0.02), BnMn (*ρ *= 0.44; *p *< 0.01), and MnCEI_TOT_ (*ρ *= 0.44; *p *< 0.01). In adjusted regression models, MnCEI_16 _was significantly associated with BnMn (β = 0.03; 95% confidence interval = 0.001, 0.05); no other biomarkers were associated with MnCEI. This suggests BnMn may be a useful biomarker of the previous 16 years of Mn exposure, but larger studies are recommended.

## 1. Introduction

Manganese (Mn) is a widely-used metal in modern industries, including mining, welding, smelting, ore processing, ferroalloy steel production, dry-cell battery manufacturing, and pesticide manufacturing. Occupational overexposure to Mn can result in postural tremor [1], decreased visuomotor coordination [2], impaired memory [3,4], deficits in information processing and working memory [3], olfactory impairment [5], and decreased motor function [3,6]. Severe Mn intoxication results in manganism with the symptoms similar, but not identical to, idiopathic Parkinson’s disease [7,8]. 

Cumulative exposure indices (CEIs) have been widely used to estimate long-term exposure to metals, including manganese, particularly in occupational settings. Prior studies on occupational manganese exposure have used CEIs based on a combination of work history questionnaire data (job titles, years of employment) [4,5,9,10,11,12], which can be supplemented with Mn air sampling [4,5,9,10,11,12]. Advantages of CEIs are that they can represent flexible time periods, may incorporate exposures from multiple routes of exposure, and can be applied in an individual basis; a disadvantage is that they are often reliant on self-reported work histories, which can be affected by recall bias.

Existing biomarkers for Mn include blood, urine, hair, and toenails. Blood and urine Mn concentrations have been shown to reflect exposure over the past several days [6,13,14,15,16]. Blood Mn (BMn) was found to be significantly elevated in a group of Italian ferroalloy workers [17] and in a cross-sectional study of 96 Russian welders [18] when compared to workers without known Mn exposure. In a study of San Francisco Bay bridge welders, active welders had significantly higher BMn concentrations than those who were no longer welding [5]. In both a study of 14 Chinese male welders and 9 smelters [19], as well as in a study of gas transmission pipeline welders [20], significantly elevated urine Mn was seen in occupationally exposed workers compared to controls. 

Mn levels in toenails and hair have also been suggested as potential biomarkers of Mn exposure [14,21,22]. Two recent studies report that toenail Mn is reflective of external Mn exposure in the prior 7–12 months among welders [14,23]. Hair Mn can also be reflective of several months of Mn exposure [21,22]. In a group of 47 welding school students, sampled from a longitudinal cohort study, hair Mn was associated with external air Mn concentrations [22]. In a cross-sectional study of Mn-exposed dry cell battery workers, participants with high Mn exposure had significantly higher hair Mn concentrations when compared to both low Mn-exposed and non-exposed workers [24]. However, the uses of both hair and nail Mn are limited due to a large variation among individuals [24] and the potential for external contamination.

Despite these positive correlations with Mn exposure, existing Mn biomarkers have several limitations, including a short half-life and high variability. BMn, for example, is only reflective of short-term Mn exposure due to the short half-life and high individual variability of Mn in the blood (~2 h–40 days) [16,25]. In the same study of dry cell battery workers conducted by Bader et al., urine Mn was unable to distinguish between Mn-exposed workers and controls [24]. Urine Mn also has inconsistency in studies that assess the association between the urinary Mn biomarker and external air Mn concentrations [14,26] since only 1% of absorbed Mn is found in urine [27].

Bone Mn (BnMn) has been suggested as a viable biomarker for cumulative Mn exposure because of Mn’s extensive accumulation in human bone (40% of the total Mn body burden) [28] and for its relatively long half-life (~8–9 years) in bone [29]. BnMn can be quantified using in vivo neutron activation analysis (IVNAA) technology, a concept originally developed and later applied by researchers from McMaster University for the assessment of Mn in the hand bones of Mn-exposed workers [30]. Researchers from this team utilized an accelerator-based IVNAA system in a feasibility study to assess BnMn in a group of 29 welders and 10 controls and found that BnMn among welders was significantly higher than that of controls [31]. In the same study, these researchers showed BnMn was significantly associated with a cumulative exposure index of participants’ lifetime occupational Mn exposure. However, despite the conceptual feasibility of using IVNAA for BnMn assessment, the large equipment is immovable, which limits the application of this novel technique.

Over the past eight years, our research team has developed a compact neutron generator-based transportable IVNAA system that can be used to assess Mn in hand bones [32,33]. Our team has previously reported on BnMn in a pilot study [34] and differences in BnMn and BMn by exposure group within a cross-sectional study of Chinese workers [35]. However, these analyses lacked a rigorous measurement of cumulative Mn exposure for each individual in the study; this assessment is needed to firmly establish whether BnMn is useful as a cumulative measure of exposure. Therefore, in this analysis, we develop two cumulative exposure indices for manganese exposure and compare these with BMn, fingernail Mn (FMn), and BnMn measurements. Our hypothesis was that a cumulative exposure index of Mn would be correlated with BnMn to a greater degree than FMn or BMn.

## 2. Materials and Methods 

### 2.1. Study Population

Sixty-one male workers from Zunyi, China were recruited for this cross-sectional study. Zunyi is colloquially referred to as “the manganese capital” of China and is home to numerous Mn-related industries. Thirty participants were recruited from an equipment manufacturing and installation factory (“manufacturing”), with limited use of Mn, and thirty-one participants were recruited from a ferroalloy factory, where manganese is extensively used. Individuals were excluded if they had any active neurological or psychiatric diseases, any known motor or cognitive impairment that was not directly related into Mn exposure, or if they did not complete the BnMn measurement. One ferroalloy worker was excluded from the study due to a lack of BnMn data, leaving 60 study participants for analyses. Five (*N* = 5) participants did not provide a sufficient quantity of fingernail sample for analyses but did complete BnMn measurements. Therefore, the population was reduced for analyses involving FMn (*N* = 55). 

Participants were brought to Zunyi Medical College on one weekday to complete all of the study protocols; participants did not work on this day. Participants completed a questionnaire, provided blood and fingernail samples, and completed a bone Mn scan. Study protocols and questionnaire responses were translated into Mandarin then back-translated to English to check the accuracy of the data prior to use. Age and years of education attained were collected from participants’ completed questionnaires. The Purdue Biomedical Institutional Review Board and Zunyi Medical College Ethical Review Board approved this study. Participants signed an informed consent document prior to their participation.

### 2.2. Cumulative Exposure Index

Work history and demographic information were collected using a questionnaire. Participants reported their job title, employer, and dates of employment for current and past jobs. These data were used to create two cumulative occupational Mn exposure indices (MnCEI) using methods adapted from prior literature [36,37]. One index included the entirety of each participant’s work history (MnCEI_TOT_). The second incorporated jobs held after 1 January 2000 (MnCEI_16_); this was an average of 16.1 (SD: 0.2) years prior to the study date. Prior research suggests BnMn has a half-life of 8–9 years [38]; thus the MnCEI_16_ was designed to reflect approximately two half-lives for BnMn.

To calculate the MnCEI, past and current jobs were grouped into similar job types, using 20 occupational groups defined by the United States Bureau of Labor Statistics [39]. Prior studies with data on air Mn concentrations for different occupations [13,40,41,42,43,44] were used to inform placement of each job into a Mn exposure rank (high/medium/low). Details of these classifications are presented in Table A1. Both the MnCEI_TOT_ and MnCEI_16 _were calculated as

MnCEI = ∑ rank_n_Y_n,_ (1)

where n = job, rank = exposure rank (high = 3, medium = 2, low =1), and Y = years employed in the job. The difference in calculation of the two CEIs is that for MnCEI_TOT_, all reported jobs were included and for MnCEI_16_, only jobs held after 1 January 2000 were included.

### 2.3. Biological Samples and Determination of Mn Biomarkers

Trained clinical staff collected whole blood samples from participants during their study visit using standard protocols. Trace metal-free vacutainers (Becton-Dickinson, Franklin Lakes, NJ, USA) were used to limit external metal contamination. Samples were frozen at −20 °C and stored until analysis. Whole blood samples were analyzed for Mn at the Chinese Centers for Disease Control and Prevention in Beijing using inductively-coupled mass spectrometry (ICP-MS); these methods have been described previously [45,46]. Briefly, blood samples were diluted with 0.01% Triton-X-00 (Sigma-Aldrich, Saint Louis, MO, USA) and 0.5% ultrapure concentrated nitric acid (Merck, Darmstadt, Germany). Samples were vortexed and then analyzed using XSERIES 2 ICP-MS (Thermo Fisher, Waltham, MA, USA). The SeronormTM Trace Elements Whole Blood Control Level 1 (Sero AS, Billingstad, Norway) was used for internal quality insurance. None of the collected samples had a Mn concentration below the detection limit (DL) of 0.11 μg/L.

Although toenail Mn is more commonly used than fingernail Mn, FMn has been used in previous studies as a biomarker of exposure [47]. Fingernails were used in this study due to a concern that toenails would have a greater potential for external contamination because participants were observed to wear open-toed shoes to the factory. Participants were asked to thoroughly wash their hands with soap to remove external debris. Fingernail samples from participants’ 10 fingers were collected using a titanium dioxide nail clipper. Fingernail samples were stored in small Ziploc bags and kept at room temperature until analysis. Samples were cleaned twice using ultrasonic cleaning procedures in 1% Triton X-100 solution (Sigma-Aldrich Inc., Saint Louis, MO, USA) [48]. Following each cleaning, the nails were rinsed multiple times with deionized (DI) water and then dried at 60 °C. After the second round of cleaning, the fingernail samples were digested at 200 °C in ultrapure nitric acid (Sigma-Aldrich Inc., Saint Louis, MO, USA), then analyzed for Mn using the ELEMENT-2 mass spectrometer (ThermoFinnigan, Bremen, Germany) at Purdue University’s Campus-Wide Mass Spectrometry Center. Mn concentrations were corrected for systematic error using an internal standard run simultaneously with the samples. The DL for fingernail Mn ranged from 1.31 to 3.97 ppb. Seventeen (28.3%) FMn measurements were below the DL but still had detectable concentrations which were larger than blank samples. Replacement of values <DL is a common method to account for the uncertainty of these samples; however, this is an unbiased method only when a small percentage of values are <DL. Instead, we chose to retain the values which were detected but <DL instead of replacing them with a constant, as has been done previously [49,50]. While these values likely have a greater variability and uncertainty compared to values >DL, this approach is likely to reduce the potential for left-censoring to induce bias in our results.

After fingernails were cut but prior to the BnMn measurement, participants were asked to wash their right hand and lower arm with soap and water for a second time. A trained research assistant then cleaned participants’ right hand and lower arm with 50% alcohol wipes. Each participant’s right hand was irradiated for 10 min to excite the ^55^Mn atoms in the hand bone to ^56^Mn. A bag filled with water was wrapped around the participant’s arm to hold it in place as well as reduce the whole body effective radiation dose (estimated at 23 µSv) [32]. After 5 min of rest to allow for the initial decay of unstable isotopes, participants then moved to a high purity germanium (HPGe) detection system that collected Mn γ ray (847 keV) spectra over the course of an hour as the ^56^Mn neutrons de-excite [32]. BnMn concentrations were calculated from the Mn γ ray spectra using a pre-existing calibration line created from a set of Mn-doped bone-equivalent hand phantoms. A Mn/Ca γ ray ratio was used to account for variation in neutron flux, hand palm attenuation, and counting geometry. This also accounts for bone density, making this a comparable measure to BnMn presented in µg/g Ca. BnMn in µg/g multiplied by 3.94 is equivalent to BnMn in µg/g Ca [33,34]. The transportable IVNAA system has a DL of 0.64 µg Mn per g dry bone (ppm) [33]. There were 19 (31.7%) participants with BnMn < DL; 13 of the 19 (68.4%) were negative values. Negative BnMn measurements can be estimated when the true BnMn value is close to zero: this has also been seen in bone lead measurements [51]. Similar to the rationale described above for retaining fingernail Mn measurements <DL in analyses, several studies of bone lead recommended retaining negative concentrations and positive concentrations which are <DL in analyses as this provides less bias and greater efficiency in comparing measures of central tendency [51,52]. Therefore, these values were included in the current analysis.

### 2.4. Statistical Analyses

All statistical analyses were completed using Stata 13.1 (College Station, TX, USA). A *p*-value ≤ 0.05 was considered statistically significant. We completed an *a priori* power calculation to determine that we needed at least 25 participants per group in order to be able to detect a difference in the BnMn of 0.5 ppm. As Pejović-Milić et al. reported a 0.9 ppm difference between welders and controls [31], we concluded that our planned sample size was adequate to detect differences in BnMn.

A review of self-reported work history revealed that most participants have held numerous jobs over their adult working life; roughly a third of participants currently employed at the manufacturing facility reported working at a Mn-related industry at an earlier point in time. Since a key aspect of this analysis is focused on cumulative Mn exposure, most analyses in the current study are based on individual Mn exposure concentrations rather than group analysis by factory, as analysis by factory may not reflect the nuanced work history of the participants. The current factory was still used as a covariate in regression analyses to account for any population differences resulting from the study recruitment strategy.

Mn biomarker concentrations were lognormally distributed; therefore, summary statistics are presented as medians and interquartile ranges (IQRs) and a natural logarithm transformation of these variables were used in analyses. A constant of 5.99 was added to all BnMn concentrations to ensure all values were positive prior to the transformation [53]. This method would affect measures of central tendency for BnMn but not its correlations or associations with other variables. Analyses were also conducted using biomarkers classified as tertiles.

Descriptive statistics included the determination of mean and standard deviation (or median and IQRs). Student’s *t*-tests were used to compare variables by factory. Spearman correlation coefficients were determined for the biomarkers and MnCEIs. Scatter plots with linear regression fit lines were created to show the unadjusted relationship between MnCEIs and the Mn biomarkers. Unadjusted and adjusted linear regression models were created to determine the association between MnCEIs and Mn biomarkers. Mn biomarkers were modeled as natural-log transformed continuous variables and as tertiles; tertiles were included as an ordinal variable. Covariates included in adjusted models were age (continuous), education (continuous), and the factory of employment (manufacturing/ferroalloy).

## 3. Results

Table 1 shows summary measures for age, education, duration of the current job, MnCEI_TOT_, MnCEI_16_, and Mn biomarkers stratified by current factory. Age, education, the duration of the current job, and MnCEI_TOT_ were not significantly different by factory. MnCEI_16_ and all Mn biomarkers were higher among current workers at the ferroalloy factory versus the manufacturing factory, these differences were statistically significant for MnCEI_16_ (*p *= 0.01), BMn (*p *= 0.02), FMn (*p *< 0.01) and was of borderline statistical significance for BnMn (*p *= 0.08). The range of Mn biomarkers was 8.43 to 39.70 μg/L for BMn, 0.15 to 935.65 μg/g for FnMn, and −5.00 to 43.02 μg/g for BnMn. 

Spearman correlation coefficients for measures of Mn exposure are reported in Table 2. MnCEI_TOT_ was only significantly correlated with MnCEI_16 _(*p* ≤ 0.01). MnCEI_16 _was significantly associated with FMn (*p* = 0.02) and BnMn (*p* ≤ 0.01). BnMn and FMn were also significantly associated with each other (*p* < 0.01). Correlations of BMn with FMn (*p *= 0.09) and MnCEI_16 _(*p *= 0.09) were approaching statistical significance.

**Figure 1:**
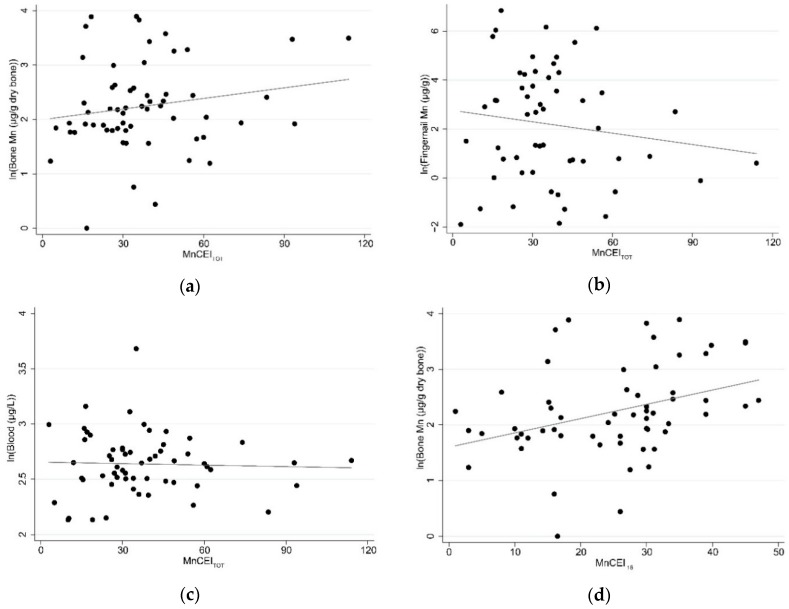

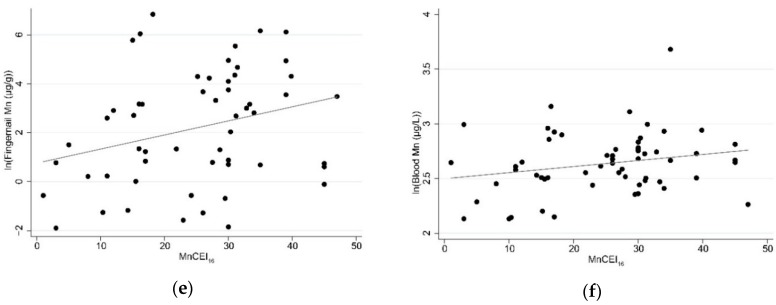
The scatterplots and unadjusted regression lines for (**a**) MnCEI_TOT_ versus bone Mn; (**b**) MnCEI_TOT_ versus fingernail Mn; (**c**) MnCEI_TOT_ versus blood Mn; (**d**) MnCEI_16_ versus bone Mn; (**e**) MnCEI_16_ versus fingernail Mn; (**f**) MnCEI_16_ versus blood Mn. Mn = manganese; MnCEI_TOT_ = Manganese cumulative exposure index for lifetime work history; MnCEI_16_ = Manganese cumulative exposure index since 2000.

Figure 1 shows scatter plots and unadjusted linear regressions for transformed Mn biomarkers compared to MnCEI_TOT_ and MnCEI_16_. MnCEI_TOT_ has a positive, but not statistically significant, unadjusted association with ln(BnMn) (β = 0.006 95% confidence interval (CI) = −0.003, 0.02). However, MnCEI_TOT_ had a negative unadjusted association with both FMn (β = −0.01; 95% CI = −0.05, 0.01) and BMn (β = −0.0004; 95% CI = −0.004, 0.003); both of these relationships were not significant. MnCEI_16 _had a statistically significant positive association with ln(BnMn) (β = 0.03; 95% CI = 0.008, 0.04) and FMn (β = 0.06; 95% CI = 0.004, 0.11); and a positive association with BMn (β = 0.005; 95% C.I = −0.0007, 0.01) which was not statistically significant.

Adjusted associations between MnCEIs and Mn biomarkers are displayed in Table 3. Results for models using continuous measures of Mn biomarkers (continuous models) were very similar to those using the tertiles of Mn biomarkers (ordinal models). An increase in BnMn was associated with an increase in MnCEI_TOT_ whereas an increase in FMn or BMn was associated with a decrease in MnCEI_TOT_. However, no models predicting MnCEI_TOT_ were statistically significant. On the other hand, increasing BnMn, FMn, or BMn were all associated with an increase in MnCEI_16_; this association was only statistically significant for the association between BnMn with MnCEI_16_. 

## 4. Discussion

In this study, we successfully developed two Mn cumulative exposure indices for our study population: one assessing lifetime Mn exposure, and one assessing Mn exposure over the prior 16 years. We hypothesized that the correlation between MnCEIs would be stronger with BnMn compared to either FMn or BMn. Our results for MnCEI_16_ supported our hypothesis. The highest Spearman correlation coefficient between a CEI and Mn biomarker was observed between MnCEI_16_ with BnMn (Spearman’s *ρ* = 0.43; *p* < 0.01); similarly, in regression models adjusted for age, education, and factory, only the association of BnMn with MnCEI_16_ was statistically significant (β = 0.03; 95% CI: 0.001, 0.05). However, in contrast to our hypothesis, we did not observe a significant association of BnMn with MnCEI_TOT_. Our results suggest that BnMn may be a feasible biomarker for exposure over the prior 16 years, but not necessarily for lifetime exposure. 

BnMn concentrations in this study were higher than previously reported values of BnMn in occupationally exposed individuals [31,34]. In the current study, the median BnMn was 2.6 µg/g bone, or 10.2 µg/g Ca when converting dry bone to Ca (there is 1 g of Ca in 3.94 g of dry bone in a reference man). These values were higher than BnMn previously reported in our pilot study of Mn-exposed workers and controls (0.66 µg/g dry bone) [34] as well as mean BnMn of occupationally exposed welders (2.9 µg/g Ca) reported by Pejović-Milić et al. [31]. There was also a wider range of BnMn concentrations in our study population (−5.0 to 43.0 µg/g bone, equivalent to −19.7 to 169.4 µg/g Ca) than in the study of welders and controls by Pejović-Milić et al. (−3.8 to 9.1 µg/g Ca) [31].

In contrast, both BMn and FMn concentrations in this study are similar to values reported previously. Median BMn in this study (14.1 µg/L) fell within the range of BMn (0–50 µg/L) reported in the recent review of the use of BMn in occupational studies [54]. Our observed FMn of 13.5 µg/g is somewhat higher than FMn values reported among a Brazilian community living near the ferromanganese industry (6.9 µg/g) [47]; this is not surprising as an occupationally exposed population would be expected to have higher exposure compared to a population with environmental exposure. Studies among United States welders have reported median toenail Mn concentration of 0.81 µg/g [55] and mean toenail Mn concentration of 6.87 µg/g [23]. Both measures are lower than our results; this difference could be due to the different occupations (welding versus ferroalloy worker) or the different matrix (toenail versus fingernail). Prior work suggests toenail Mn is associated with the prior 7–12 months of exposure; it is less clear what this period would be for fingernails [14]. Fingernails grow faster than toenails, which would suggest a shorter half-life for fingernail concentrations; however, toenails are generally shorter than fingernails, which might effectively reduce the influence of growth rate on half-life [56]. A recent study by Sakomoto et al. evaluated mercury concentrations in fingernails, toenails, and hair; they report very high correlations between fingernails with toenails, and both nails with hair collected 3–4 cm from the scalp, suggesting that fingernail and toenail samples represent similar exposure periods [56]. 

There are few studies which have compared CEI measures with Mn biomarkers. In a study of 58 ferroalloy Italian factory workers, Lucchini et al. reported a significant positive association of lifetime Mn CEI with both BMn [12]. In the current analysis, although the results were not significant, we observed a negative association of lifetime CEI with blood Mn, but we did observe a nonsignificant positive association of BMn with MnCEI_16_. In the Lucchini et al. study, the workers were sampled after a period of temporary layoff from work, whereas in the current study workers did not work during the day they were sampled but likely worked the day before. As blood Mn concentrations have been shown to vary substantially over the course of a week or even within the same day [14], this difference in methodology might explain the difference in results.

We are aware of only one other study which compared BnMn with a cumulative exposure index, conducted in Ontario, Canada [31]. Pejović-Milić et al. collected data on occupational history and used IVNAA to quantify BnMn from 28 welders and 8 controls. This study reported that BnMn was significantly associated with a lifetime CEI for Mn [31]. Although similar to our results in that they reported that BnMn is associated with a CEI, in our study we only observed the association between the prior 16 years of exposure, not lifetime exposure. Although it is impossible to definitely identify the cause for this discrepancy, it is possible that this may be related to a higher job turnover among the Chinese workers. As noted earlier, we observed greater job turnover than expected within this group of Chinese workers; therefore, our estimates of a MnCEI_TOT _versus MnCEI_16_, although correlated, clearly represent different patterns of exposure and correlations with BnMn. In contrast, our experience in the United States is that welders generally have less job turnover compared to other factory workers, as they tend to be better paid and in high demand. If this is also true in Canada, it is plausible that in the population observed by Pejović-Milić et al., lifetime work history may also be highly correlated with cumulative exposure over the past 16 years. 

This study has some weaknesses. The sample size is relatively small. A *post hoc* power calculation suggested that we had sufficient power to detect an association of BnMn with MnCEI_16_, but not necessarily with MnCEI_TOT_. Thus, while we have confidence in our results for MnCEI_16_, it is possible that our inability to observe an association of BnMn with MnCEI_TOT_ could be due to the small sample size of this study. Additionally, participants reported their exposure history which, in some cases, spanned decades. This could have resulted in some recall bias. However, to minimize the potential for recall bias, we relied on more general information, that is, job titles and the length of employment instead of finer details which may be more difficult to recall accurately, that is, specific job tasks and the hours spent doing each task per week. 

Several studies incorporating cumulative exposure indices of Mn exposure incorporate air sampling data from the factory being studied in order to help confirm the accurate placement of individuals into the CEI [4,5,10]. A limitation of this study was that we did not have air sampling data to assist with the CEI assessment; however, as demonstrated by prior studies, it is possible to construct a CEI based on occupational history [36,37]. Although we lacked air sampled from our participants’ workplace, we did refer to literature linking specific jobs with air Mn concentrations where it was possible to increase the rigor of our classifications (Table A1). While the CEI calculation in this analysis is not able to provide precise exposure estimates, it is still sufficiently detailed to rank our participants’ Mn exposure. 

There are several strengths of this study. Mn biomarkers were collected and analyzed using state of the art methods with high precision. We additionally incorporated numerous protocol elements to reduce the potential for external contamination of FMn and BnMn samples: participants washed their hands multiple times, and post-collection fingernails were cleaned, twice, with a thorough protocol. Study questionnaires were originally developed in English, translated into Mandarin for this population, and then back-translated into English by different study staff to ensure the consistency and quality of the translation. Study staff fluent in both Mandarin and English administered the study questionnaire and translated the responses data back to English. Additionally, several study staff members are fluent in both English and Mandarin.

There is still very limited evidence regarding cumulative biomarkers of Mn exposure; thus a significant strength of this study is our use of bone manganese. Prior work has introduced CEIs which reflect different time periods [14,23]; our incorporation of two CEIs is also a strength of this analysis as it allowed us to expand upon prior work by demonstrating differences in Mn biomarker-CEI associations based on exposure duration. 

## 5. Conclusions

The current study quantified a novel Mn biomarker, BnMn, and developed cumulative exposure indices for a lifetime and the past 16 years of occupational Mn exposure. The 16-year CEI was significantly associated with bone manganese concentrations. These results suggest BnMn could be a useful biomarker of cumulative exposure among populations with occupational Mn exposure is warranted; however, our results should be followed up with larger studies.

## Figures and Tables

**Table 1:** The means (standard deviations) for the selected population characteristics stratified by factory.

Characteristic	Manufacturing Factory (*N* = 30)	Ferroalloy Factory (*N* = 30)	Total Population (*N* = 60)
Age, years	48.2 (9.4)	46.7 (6.1)	47.3 (7.9)
Education, years	10.9 (4.0)	9.1 (3.6)	10.0 (3.9)
Duration of current job, years	9.5 (8.7)	8.4 (4.1)	9.0 (6.8)
MnCEI_TOT_, no units	41.2 (28.1)	33.9 (13.0)	37.5 (22.0)
MnCEI_16_, no units	21.2 (12.5) *	28.7 (8.7) *	25.0 (11.3)
Blood manganese, µg/L	13.4 (3.1) *^,1^	15.2 (5.9) *^,1^	14.1 (4.0) ^1^
Fingernail manganese, µg/g	1.5 (1.9) *^,1,2^	42.8 (116.2) *^,2,3^	13.5 (58.5) ^1,4^
Bone manganese, µg/g	0.9 (4.5) ^1^	3.0 (16.3) ^1^	2.6 (7.2) ^1^

MnCEI_TOT_ = Manganese cumulative exposure index for lifetime work history; MnCEI_16_ = Manganese cumulative exposure index since 2000. * Student’s *t*-test* p *< 0.05 for difference by factory; ^1^ Median (interquartile range) is presented and natural logarithm is used in *t*-tests; ^2 ^*N* = 26; ^3 ^*N* = 29; ^4 ^*N* = 55.

**Table 2:** The Spearman’s ρ (*p*-value) for correlations among manganese biomarkers and cumulative exposure indices, *N* = 60.

Variable	Blood Mn	Fingernail Mn	Bone Mn	MnCEI_TOT_	MnCEI_16_
Blood Mn	1.00 (1.00)	--	--	--	--
Fingernail Mn	0.23 (0.09) ^1^	1.00 (1.00) ^1^	--	--	--
Bone Mn	0.16 (0.23)	0.45 (<0.01) ^1^	1.00 (1.00)	--	--
MnCEI_TOT_	−0.01 (0.97)	−0.13 (0.35) ^1^	0.16 (0.22)	1.00 (1.00)	--
MnCEI_16_	0.22 (0.09)	0.32 (0.02) ^1^	0.43 (<0.01)	0.66 (<0.01)	1.00 (1.00)

Mn = manganese; MnCEI_TOT_ = Manganese cumulative exposure index for lifetime work history; MnCEI_16 _= Manganese cumulative exposure index since 2000. ^1 ^*N* = 55.

**Table 3:** The β (95% confidence interval) for MnCEI_TOT_ or MnCEI_16 _in adjusted regression models predicting manganese biomarkers, *N* = 60.

Mn Biomarker	Model ^1^	MnCEI_TOT_	MnCEI_16_
Blood Mn	Continuous	−0.0003 (−0.004, 0.003)	0.002 (−0.005, 0.009)
	Ordinal	−0.0008 (−0.02, 0.02)	0.008 (−0.04, 0.05)
Fingernail Mn ^2^	Continuous	−0.007 (−0.03, 0.01)	0.004 (−0.03, 0.04)
	Ordinal	−0.008 (−0.04, 0.02)	0.009 (−0.05, 0.06)
Bone Mn	Continuous	0.007 (−0.003, 0.02)	0.03 (0.001, 0.05) *
	Ordinal	0.02 (−0.0003 , 0.05)	0.08 (0.03, 0.14) *

Mn = manganese; MnCEI_TOT_ = Manganese cumulative exposure index for lifetime work history; MnCEI_16_ = Manganese cumulative exposure index since 2000. * *p* < 0.05 ^1^ Continuous models use ln(Mn biomarker); ordinal models use biomarker tertiles; ^2^ *N* = 55.

## Appendix A

**Table A1:** The classification of job titles into exposure ranks used in cumulative exposure index (CEI) calculation.

Job Title ^1^	Occupational Category ^2^	Air Mn ^3^	Exposure Rank
Welder/arc welder	Metal ore processing	Welders: 0.01 to 2.13 mg/m^3 ^[40]; Ferroalloy workers: 0.098 to 0.374 mg/m^3^ [13]	3: high (direct exposure)
Smelter			
Ore combustion			
Ore extraction			
Manganese ore processing			
Underground Mn ore mining			
Manganese electrolysis			
Director	Management (ferroalloy)	Ferroalloy workers (indirect exposure) = 0.01–0.11 mg/m^3 ^[13]; Mining = 0.21 mg/m^3 ^[41]; Die-casting foundry = up to 0.14 mg/m^3 ^[43]	2: medium (indirect exposure)
Manager/Project manager			
Ore production manager			
Equipment management			
Driver	Transportation and material moving (Ferroalloy)		
Driver: ore transporting			
Trainman			
Metal product assembler	Assemblers and fabricators		
Maintenance	Installation maintenance and repair (Ferroalloy)		
Equipment repair			
Machine repair			
Cleaner	Building grounds and cleaning (Ferroalloy)		
Machine operator	Non-ferrous metal production and processing		
Miner	Mining		
Die-casting hardware	Molding		
Refinery	Metal-refining furnace operators and tenders		
Lime processing	Lime product manufacturing		
Factory/general worker	Other occupations (ferroalloy) ^4^		
Wireman			
Advertisement	Advertisement and marketing	Auto repair: 0.00045 mg/m^3 ^[38]; Administrative Support: 0.001 mg/m^3 ^[44]	1: low (low/no exposure)
Marketing			
Driver	Transportation and material moving (manufacturing)		
Stevedore			
Secretary	Administrative support		
Security guard/personnel	Protective services		
Machine repair	Installation, maintenance, and repair (manufacturing)		
Weeding	Building and grounds cleaning (manufacturing)		
Designer	Industrial designers		
Car repair	Automotive repair and maintenance		
Cement factory worker	Construction		
Construction worker			
Road construction/roadman			
Brick carrier			
Manager	Management (manufacturing)		
Vegetable storage manager	Other occupations (manufacturing/other employer) ^4^		
General/other worker			
Private business			
Wireman			

^1^ As reported by participants; ^2^ Occupational category encompassing groups of jobs with similar responsibilities, these are based on US Department of Labor definitions [39] and current factory; ^3^ Previously reported air Mn concentrations for jobs in a specific occupational category; ^4 ^Job titles did not match a U.S. Department of Labor category.
